# Motivations for using misoprostol for abortion outside the formal healthcare system in Colombia: a qualitative study of women seeking postabortion care in Bogotá and the Coffee Axis

**DOI:** 10.1186/s12978-024-01814-0

**Published:** 2024-06-01

**Authors:** Juliette Ortiz, Nakeisha Blades, Elena Prada

**Affiliations:** 1 Independent consultant and former Principal Research Associate of Fundación Oriéntame, Bogotá, Colombia; 2 Division of Research, Guttmacher Institute, New York, NY USA; 3 Independent consultant, Santa Marta, Colombia

**Keywords:** Misoprostol, Self-managed abortion, Legality perception, Post-abortion care, Misoprostol, Aborto autoinducido, Percepción de legalidad, Atención postaborto

## Abstract

**Background:**

In 2006, a Constitutional Court ruling partially decriminalized abortion in Colombia, allowing the procedure in cases of rape, risk to the health or life of the woman, and fetal malformations incompatible with life. Despite this less prohibitive law, some women and pregnant people preferred self-managing their abortions outside the formal healthcare system, often without accurate information. In 2018, we undertook a study to understand what motivated women to self-manage using medications that they acquired informally. Colombia has since adopted a progressive law in 2022, permitting abortion on request through the 24th week of pregnancy. However, the implementation of this law is still underway. Examining the reasons why women chose to informally self-manage an abortion after 2006 may not only highlight how barriers to legal services persisted at that time, but also could inform strategies to increase knowledge of the current abortion law and improve access to services going forward.

**Methods:**

In-depth interviews were conducted in 2018 with 47 women aged 18 and older who used misoprostol obtained outside of health facilities to induce an abortion, and who were receiving postabortion care in two private clinics. Interviews explored what women knew about the 2006 abortion law which was then in effect, and the reasons why they preferred informal channels for abortion care over formal healthcare services.

**Results:**

Women’s motivations to use misoprostol obtained outside the formal healthcare system were influenced by lack of trust in the healthcare system along with incomplete and inaccurate knowledge of the abortion law. Conversely, women considered misoprostol obtained outside the healthcare system to be effective, affordable, and easier to access.

**Conclusions:**

Obtaining misoprostol outside the formal healthcare system offered a more accessible and appealing prospect for some women given fears of legal repercussion and stigma toward abortion. Though this preference will likely continue despite the more liberal abortion law, strategies should be implemented to broaden knowledge of the recent change in law and to combat misinformation and stigma. This would support knowledge of and access to legal abortion for those who wish to avail themselves of these services.

**Supplementary Information:**

The online version contains supplementary material available at 10.1186/s12978-024-01814-0.

## Abstract (plain language summary)

Despite the availability of legal services, pregnant individuals in Colombia have continued to seek abortion outside of the formal healthcare system. Understanding their knowledge of the abortion law and their perceptions of legal abortion services may provide insight into what motivates them to seek alternative routes of abortion.

Women who obtained misoprostol outside of the formal healthcare system were interviewed in 2018, twelve years after abortion was partially decriminalized in Colombia (and prior to the full decriminalization of abortions up to 24 weeks in 2022). A combination of factors led women to seek misoprostol in this manner: incomplete knowledge of the abortion law, fear of legal consequences, fear of social stigma, and a desire for a faster and private process.

Self-management with informally obtained medication can offer pregnant individuals the opportunity to have an abortion on their own terms, especially when abortion in the formal healthcare system appears to be unavailable, proves difficult to access, or is accompanied by social and legal risks. While increasing awareness that abortion is legal in Colombia might empower pregnant people to seek formal care, judgment from their community, healthcare providers, and law enforcement may still serve as powerful hindrances to obtaining these services unless there are efforts made to combat abortion stigma as well.

**Supplementary Information:**

The online version contains supplementary material available at 10.1186/s12978-024-01814-0.

## Abstract - Español

**Contexto:**

En el 2006 la Corte Constitucional de Colombia despenalizó el aborto en casos de violación, riesgo para la salud o vida de la mujer y malformaciones fetales. A pesar de ello, algunas mujeres y personas con capacidad de gestar continuaron obteniendo abortos fuera del sistema de salud. En el año 2018, realizamos un estudio para comprender qué las motivaba a hacerlo.

Desde entonces Colombia ha adoptado un marco legal más progresista, permitiendo en el año 2022 el aborto a solicitud hasta la semana 24 de embarazo. Sin embargo, la implementación de este marco legal aún está en curso. Examinar las razones por las que las mujeres recurrieron a auto inducirse un aborto de manera informal después del 2006 no solo informa sobre las barreras a los servicios legales en ese periodo, sino también ayuda a desarrollar estrategias para aumentar el conocimiento del marco legal actual y mejorar el acceso a los servicios

**Métodos:**

Durante el 2018 entrevistamos a 47 mujeres mayores de 18 años que compraron misoprostol fuera del sistema de salud para inducirse un aborto, y que recibieron atención postaborto. En las entrevistas exploramos lo que sabían sobre el marco legal de ese momento –el del 2006–, y las razones por las cuales recurrieron a fuentes informales para abortar.

**Resultados:**

Las motivaciones de las mujeres para usar misoprostol obtenido de fuentes informales resultan de la desconfianza hacia el sistema de salud y un conocimiento incompleto del marco legal. En contraste, las mujeres consideran que el misoprostol obtenido por fuentes alternativas ofrece eficacia y asequibilidad, y es más fácil de obtener.

**Conclusiones:**

Obtener misoprostol fuera del sistema de salud formal es una alternativa más llamativa para algunas mujeres dadas las preocupaciones sobre las repercusiones legales y el estigma asociado al aborto. Aunque esta preferencia persista a pesar del cambio del marco legal, se deben implementar estrategias para ampliar el conocimiento sobre la despenalización y combatir la desinformación y el estigma. Esto garantizaría que las mujeres estén informadas y puedan acceder a servicios legales de aborto de calidad si así lo desean.

## Introduction

In February 2022, the Constitutional Court of Colombia completely decriminalized abortion up to 24 weeks of gestation [1]. Prior to this decision, in May 2006, the Constitutional Court made abortion permissible under three circumstances through ruling C-355: if the pregnancy represented a threat to the life or (mental or physical) health of the woman; was the result of rape or incest; or when there were fetal malformations incompatible with life [2]. To-date, these grounds continue to determine access to abortions occurring after 24 weeks of gestation.

After the 2006 ruling, feminist and civil society organizations promoted awareness of the new legal options. They hosted workshops, lobbied the government to explicitly outline the guidelines for service provision, and provided technical assistance to the Ministry of Health as policies for health insurance companies and health providers regarding service delivery were fleshed out [3, 4]. Between 2006 and 2021, pregnant individuals requesting legal abortion care were required to report to either a physician or psychologist and indicate which of the three permitted circumstances applied to their situation [5]. For individuals soliciting an abortion under the health and fetal malformation exemptions, the health professional’s approval determined their access to care [5]. Those requesting abortion under the rape exemption had to present a filed police complaint to the healthcare provider.

Abortion also became part of the basic universal health plan, thus allowing individuals access to legal services at public sector facilities regardless of whether they had health insurance. Furthermore, private facilities providing abortion care entered into agreements with some health insurance companies, allowing for patients insured with those companies to access comprehensive abortion care at no cost. The Court also established that the process of obtaining a legal abortion should not take more than five days and that individuals are guaranteed the right to freely decide to have an abortion throughout their care pathway [5]. 

Despite the establishment of these processes to facilitate access to legal abortion care, pregnant people still faced obstacles. These obstacles stem in part from conservative backlash to the 2006 decision. Religious organizations, anti-abortion groups, as well as key figures in government launched various efforts aimed at sabotaging the ruling or forcing its reversal [6, 7]. More recently, representatives of the conservative movement have filed various lawsuits focused on protecting life from conception, with the goal of forcing the Constitutional Court to criminalize abortion once again [8]. Though these efforts were not successful, they signaled a strong rejection of abortion rights among some sectors of Colombia’s population.

Healthcare providers also presented barriers. A study of abortion attitudes among key informants and physicians in Bogotá in 2014 identified a spectrum of attitudes toward providing referrals to women soliciting legal abortion care [9]. At the more extreme end of the spectrum, providers indicated absolute opposition to abortion, often refused to provide referrals (despite an obligation to do so), and saw it as a medical and moral responsibility to prevent abortions from taking place [9]. Even providers whose objections were less extreme suggested that they might try to dissuade their patients from obtaining abortions, or would refuse to provide care based on their own determinations of whether abortion was appropriate for the situation [9]. 

Likewise, some health professionals have been found to employ a narrow interpretation of the health exception, refusing to provide services unless the patient is sick or dying [10, 11]. Stanhope and colleagues [11] also found that some physicians imposed unnecessary requirements, such as requiring partner consent. In addition to creating obstacles, providers exercised moral judgment over their patients, prioritizing the welfare of the pregnancy over the pregnant person’s, attempting to shame them into continuing their pregnancies or to feel guilty following the procedure, often through harsh treatment and/or publicly denouncing them [12, 13]. These practices directly infringed upon the law, which states that physicians are obliged to respect people’s decisions regarding their pregnancy, and that those declaring conscientious objection must refer pregnant individuals to providers that will perform the procedure for them [5, 13]. Some health professionals were even directly implicated in the criminalization of abortion. In a study that compiled information on abortion cases investigated between 2006 and 2018 because they allegedly did not comply with the legally permissible criteria, healthcare professionals reported just over half of the cases and ultimately accounted for two-thirds of the cases that resulted in a conviction [14]. This occurred despite the fact that under Colombian law, professional secrecy is inviolable.

Women seeking legal abortions have reported stigmatizing experiences and poor treatment from medical providers, including legal threats, dismissive attitudes toward their situations, and outright rejection of care [7, 13, 15]. Venezuelan individuals who have experienced pregnancies while migrating to Colombia have also reported experiences of xenophobic discrimination [16]. Further, women have expressed fears of being judged by their family, partners, and broader social circles should their abortion seeking be exposed, anxieties that are sometimes exacerbated by their own misgivings about abortion [15, 17]. 

Confusion and lack of information have also been found to compound structural barriers to legal abortion care. In a 2012 study exploring barriers to legal services, the most noted barrier was financial; respondents in this study believed that the cost of the abortion was out of reach for them, contributing to delays in obtaining care [18]. However, as previously noted, abortion is available at no cost under the basic universal health plan, a piece of information that these respondents may have been unaware of or did not feel was an option they could employ [18]. Abortion patients have also reported that despite making appointments at legal healthcare facilities, difficulty locating these sites led them to unofficial/clandestine sites with health professionals of questionable ethics and skill [17]. Others reported that even when they did arrive at a site that provided legal services, they were unable to obtain the abortion because they did not know that some clinics were only equipped to provide abortions up to a certain gestational age [17]. Other abortion patients have reported being unaware of the availability of legal services entirely [13, 17]. Some of these patients include migrants from Venezuela, who expect that the abortion law in Colombia is as restrictive as in their country of origin, where abortion is only legally permitted to save a woman’s life [16]. Patients that have reported an awareness of the law still reported either incomplete knowledge of the law’s parameters for abortion or a misinterpretation of them that influenced how they went about pursuing access [15, 17]. 

Given the many obstacles to access in this polarized climate, it is not surprising that pregnant individuals have sought abortion outside of the formal healthcare system. In fact, recent research in Colombia finds that those pursuing abortion are still facing many of the same structural barriers as they did prior to the change in law in 2022 [19]. While national statistics on the incidence of abortion in Colombia are out of date, multiple studies have found that the incidence of postabortion care cases associated with induced abortions greatly exceeds that of induced abortion cases treated within the formal healthcare system, suggesting that most induced abortions occur outside of the formal sector [19, 20]. When Prada and colleagues [20] estimated the incidence of abortion in 2008, they calculated that about half of the induced abortions that took place that year were likely performed using misoprostol. In 2007, misoprostol was approved by the National Institution of Medication Surveillance in Colombia for legal abortion care, and made legally accessible through medical prescription [21]. However, misoprostol has been found to be sold informally without a prescription through various outlets including street sales, online sellers, and independent drugstores, among others [22]. 

Some work has touched on how misoprostol has been used in Colombia for medication abortion, but this work has only included individuals who have sought out legal abortion care [13, 15]. Prior to the decriminalization of abortion, we conducted in-depth interviews with individuals who informally obtained misoprostol to self-manage an abortion. We define ‘informal’ self-managed abortion as the acquisition of misoprostol through unofficial channels, such as drug stores or online sellers, to induce a medication abortion on their own, outside the formal healthcare system. In this paper, we examine what these participants knew of the (then) abortion law and what led them to informally self-manage their abortions rather than seek care in the formal healthcare system.

Although these data were collected before the 2022 ruling, we expect that this analysis will shed light on the barriers that hindered access to legal abortion care after the ban was partially lifted, barriers which may persist despite the current broader decriminalization of abortion. Furthermore, we anticipate these findings will contribute to a deeper understanding of the factors that may motivate pregnant people to self-manage their abortions informally rather than seeking legal services.

## Methods

This study was a collaboration between the Guttmacher Institute, a sexual and reproductive health research and policy institute based in the United States, and Fundación Oriéntame (Oriéntame), a private not-for-profit organization based in Colombia that provides legal abortion care. A participant in the development of Colombia’s guidelines for legal abortion care, Oriéntame fully complies with the national standards for abortion care provision, including providing comprehensive care without obstructions or unnecessary delays. Oriéntame’s services also include postabortion check-ups for women who want to confirm the completion of an informally self-managed abortion. If the abortion attempt was unsuccessful, Oriéntame offers postabortion care.

Ethical approval for this research was provided by the Comité de Ética en Investigación de la Fundación Oriéntame and the Institutional Review Board of the Guttmacher Institute. These data were collected as part of a larger study aimed at understanding informal access to and use of medication abortion in Colombia, Indonesia, and Nigeria. Other results from the study have been published elsewhere [21–26].

### Sample and recruitment

Throughout the following sections we will primarily refer to the participants in this study as ‘women,’ as all the individuals in our sample identified as cisgender women. However, we acknowledge that people of different gender identities may become pregnant and seek abortion. The women in this study were recruited between May and July of 2018 from two Oriéntame clinics, one located in Bogotá and the other in the Coffee Axis area. These regions were selected because they included a range of options for abortion services, spanning from informal misoprostol sales to legally recognized abortion care clinics. Both locations also have populations reporting a moderate liberal stance towards abortion rights, with 59% of the Coffee Axis residents and 69% of Bogota residents agreeing that it is important for political candidates to advocate for abortion rights in 2023. Overall, 59% of Colombia’s population agrees with this viewpoint [27]. These regions significantly differ in size, however. In 2018, the population of Bogotá was nearly three times that of the Coffee Axis [28]. 

Women 18 years of age or older, who had bought misoprostol to induce an abortion through an informal sale, and who solicited a postabortion care check-up from one of the two Oriéntame sites were eligible for participation. Potential participants were informed that a study was taking place at the clinic after they received their care and were introduced to an interviewer if they expressed interest in learning more. Those who consented to participate were interviewed on the same or the following day by one of two trained interviewers who were based at each of the clinic sites.

Interviews took place in Spanish in private rooms at the clinics and lasted on average 50 min. The interview covered the participant’s decision-making process to have an abortion, her experience accessing the medication through an informal sale to attempt to abort, and any attempts she might have made to seek additional care. We interviewed 47 women, 27 in Bogotá, and 20 in the Coffee Axis. They were provided USD 30 each to compensate them for their time, and USD 5 to cover transportation costs.

### Analysis

All interviews were recorded and transcribed verbatim into Spanish. Transcripts were checked for accuracy and de-identification by JO. We (JO, NB, EP) developed a coding structure based on the interview guide and JO and EP coded the interviews in NVivo 14 (QSR International, Melbourne, Australia) after 90% inter-coder reliability had been established. Following Miles and Huberman [29], the coded interviews were organized into matrices and then summarized into bullet points by JO, NB, and EP.

For the analysis, we focused on knowledge of the abortion law, including the number of permissible criteria each respondent could identify. We also identified themes related to why participants avoided obtaining care in formal healthcare settings and what factors influenced their decision to obtain medications through informal sales to self-manage their abortion. We present findings here using translated quotes to illustrate themes. As the themes that arose from this analysis did not differ considerably across demographic categories, these data are not presented in stratified groups.

Sample characteristics are presented in Table 1. More information regarding the recruitment and data collection for this study, along with findings on how participants accessed and used the medications they obtained, are available in a previous publication [22]. 

**Table 1:** Characteristics of the participant sample, Colombia

	*N* = 47
* Location*	
Bogotá	27
Coffee Axis	20
* Age*	
18–19	2
20–24	19
25–29	15
30–34	6
35+	5
* Relationship Status*	
Never married/Not currently cohabitating	26
Cohabiting	10
Separated/Divorced	11
* Education*	
Less than secondary school	7
Secondary school	10
Technical school (incomplete/complete)	15
College (incomplete/complete)	14
Graduate	1
* Occupation* ^*^	
Unemployed	6
Housewife	9
Student	7
Works outside home	32
* Number of previous births*	
0	20
1	15
2	12

*Respondents could select multiple options

## Results

### Respondents’ knowledge of the abortion law

Before receiving care at Oriéntame, just over half of the participants (*n* = 25/47) reported knowing at least one of the criteria for accessing legal abortion care, with thirteen of these women reporting knowledge of all three criteria. Compared to participants in the Coffee Axis, Bogotá participants were better able to identify multiple criteria. The most often reported criteria were fetal abnormality and rape. Among those who noted the health exemption, only a few (*n* = 5/15) referenced risk to the woman’s mental health as well as physical health. However, none of these respondents thought that they could have employed the mental health exemption to obtain legal abortion care. In fact, one woman who was a law student at the time of the interview, reported it was difficult to understand what constituted a mental health risk.

They talk to you at university about the three criteria to have a [legal] abortion. In cases of malformation, rape or mental risk, but you don’t think that not being prepared, not feeling well emotionally, or not having the economic resources to bring a child into the world is a [mental] risk… you think that “If I go [to a health facility] and say that I do not feel prepared, that I do not have money, and that I am not even married… they [health staff] will say ‘Well, you still have to have it…’”. You don’t think that these are reasons for which you can have an abortion. (23 years old, the Coffee Axis)

All other participants believed that abortion was completely illegal. Two of these women were Venezuelan migrants who thought that the abortion law in Colombia was as restrictive as in their country of origin.

A consequence of this incomplete knowledge about the abortion law is that some participants never considered obtaining care at a health center because they thought that abortion would not be covered through their health insurance companies, that abortions were only available under circumstances that were not applicable to them, or did not think abortion services were available at all – as was the case of women who lived in smaller towns.

If I went to a hospital or consulted the insurance company, they were going to put me into trouble or going to judge me… I thought [abortion] was illegal. Or I mean, up until now I don’t know how legal it is [to have an abortion] in Colombia, but… well, I thought that maybe at the clinics it was something illegal, to have an abortion. That’s why I didn’t go to the clinics, [and I] didn’t consult with [my health insurance company]. (34 years old, the Coffee Axis)

…where I live [a small town with no private reproductive health facilities] there are no centers like this [refers to Oriéntame]… if you are bleeding, you can go to [a public health facility], they’ll admit you to the emergency room and maybe they give you something to stop it, but they won’t [perform an abortion]… (24 years old, the Coffee Axis).

### Reasons for obtaining abortion outside of the formal healthcare system

In describing how they came to use informally acquired misoprostol to terminate their pregnancies, the women in this study primarily noted reasons why they wanted to avoid having an abortion within the formal healthcare system.

#### Fear of legal consequences

Because most women in our study either believed that abortion was completely illegal or that their situation did not fall under the permitted circumstances, fear of legal consequences played a major role in why they avoided obtaining abortion services in a formal healthcare facility. Participants expected that they could face criminal charges and that health professionals would be the ones to report them to the authorities.

It‘s scary – that the doctor will call the police and then a scandal will break out, then everybody would find out [about this abortion]. This must be done clandestinely. (32 years old, the Coffee Axis)

This anxiety meant that even when women had concerns about the physical effects or success of the abortion attempt, they were less inclined to try to obtain postabortion care. They anticipated there would be consequences if they revealed or their provider concluded that they had tried to interrupt their pregnancies. Furthermore, they perceived the health system as an extension of law enforcement.

It scares me… tell me what am I going to tell [a doctor]? It’s just that I took twelve pills to abort, and some injections, and I feel [physically] bad. What is he going to tell me? They’re going to shoot me [metaphorically speaking], [tell me] that this is illegal…I am going to have a huge legal problem. (26 years old, the Coffee Axis)

Prior to obtaining care at Oriéntame, five women sought postabortion care at a health facility. While three of the women did not disclose their abortion attempt, the other women admitted to using pills to abort. These two women fortunately did not experience negative interactions with their providers after this admission. Another woman who sought care at Oriéntame initially, described a very different experience that highlighted why participants’ fears were founded. This woman needed care beyond what could be provided at Oriéntame and was referred to another facility. She related that the healthcare personnel she encountered at this facility treated her poorly. She said she was left waiting for prolonged periods while mostly undressed, not offered any food or water, and not given information about why she was taken into care as an inpatient.

They took a blood test… and when the results came out, all of a sudden, I was hospitalized. I did not know why. They hadn’t told me whether the abortion was incomplete, that it was retained, or that I was at risk. …I stayed on my stretcher and at about six o’clock in the evening a social worker came in. She asked me what I had taken. Well, I told her that I had taken some aspirin, that’s all. I didn’t tell her anything [about taking misoprostol pills]… She told me that the only one who knew [what I had done] and could forgive me was God. Then she told me, “Look, the cops are going to come, they are going to ask you some questions and you are going to answer truthfully”. (23 years old, Bogotá)

This participant related that while she was waiting, another woman who had also taken abortion pills was placed in the same room. When the police arrived, both she and this other woman were interrogated, and the police called for backup to take both women into custody for having illegally induced an abortion. Meanwhile, she (and her sisters who had accompanied her) were called to be seen by a gynecologist who had recently arrived at the hospital. While they were in his office (which was within the hospital) the police started to look for them. The participant felt trapped because she had been earlier told that pregnant women could not leave the hospital voluntarily.

…the three of us were in the [gynecologist’s office], with the door closed. We saw about ten policemen through the window running around, looking for me… I was afraid. Five policemen came in all at once to the office after the doctor called for them. I was in my robe. I was half-naked. They piled in all at once and asked me “You’re the one with the abortion, right? You are [Participant’s name]?“. The gynecologist said, “She’s not the one who had the abortion, that’s the other one.” He said, “She came here because she was hemorrhaging.” (23 years old, Bogotá).

This participant was able to avoid arrest because of the intervention of the gynecologist. It was not clear to her why he did so, but he did tell her that it was to her “advantage” that the fetus was still alive and advised her to tell the police that she had only taken aspirin if asked. This was a traumatizing experience for the participant, who related that this took place over an 18-hour period. She later returned to Oriéntame and was referred to another hospital where her abortion was completed.

#### Fear of Ill-treatment/privacy loss

Another barrier to seeking formal care raised by participants was the fear of mistreatment from healthcare workers due to anti-abortion attitudes. Participants related anxieties that their behavior and morality would be questioned and felt that it would be difficult to request an abortion publicly.

…[Me and my partner did not think of] going to a health center and requesting this, no. Because immediately they’re not going to think of the [pregnant] person, but rather they are going to think about a baby that is going to be born. So no, we did not consider [going to a clinic for care]. (22 years old, the Coffee Axis)

Anxiety related to being judged also meant that seeking care in the formal healthcare system was not viewed as an option even when an abortion might have been obtained under one of the legal criteria. One woman, whose pregnancy resulted from rape, specifically decided not to report what had happened to her out of fear of how she would be treated.

My pregnancy was not wanted, it was not planned, and it was not my responsibility. It was against my will, but I did not want to bring the authorities into it because I would be misjudged. I was at a party and they poured something in my drink and I don’t know what happened next. I felt that if I went to the authorities they were going to say “You were at a party, that’s why you were drunk. You gave [your body] to anyone”, but it’s not like that. (26 years old, Bogotá)

As a result, a consideration for some participants was the ability to keep their abortion seeking private. Some women related that they did consider going to a health center for care, but ultimately decided against it because they thought they would have to consult multiple providers to find one that would be willing to perform the procedure, therefore compromising their privacy. Others were concerned that obtaining an abortion through the formal healthcare system would increase the likelihood that their situation would be exposed, because the procedure would be part of their medical history.

I know that in many places the hospitals do not agree with abortion, so they would not [perform it] and I kept looking. And also…the issue of fear that this would appear in my clinical records, that they will call a guardian, my dad, my mom…[I wanted] to avoid all [of] that process. (23 years old, the Coffee Axis)

Further, the healthcare centers that were most accessible to some participants living in small cities were not only located within their community, but also staffed by people that they knew. This was the case of a woman who lived in a small town in the department of Santander. While going through her abortion process, she took a job in Bogotá. After relocating, she scheduled a postabortion check-up at Oriéntame.

…the [health center] is still good there [where the respondent is from], but I would be attended by my friends’ parents –I would die with [shame if that happened]. I would have preferred to come to a health facility here in Bogotá, but I couldn’t [come at that time]. (22 years old, Bogotá)

Visiting a health facility also carried the risk of being expected to continue with the pregnancy. Participants related that if their pregnancies became known to health professionals, then they would be pushed to obtain prenatal care services, without any consideration of whether they desired to have an abortion. This could then open them up to scrutiny should they either avoid obtaining prenatal care or are later discovered no longer pregnant. One woman related that she heard that being tracked in this way could eventually lead to legal issues.

Since I found out I was pregnant, well, I had no thoughts of having the baby. And then [others] told me that if I went to the doctor or something, that they would keep track of me. That’s what they told me. And if I said that I did not want to have the baby or something, I could have legal problems because abortion is illegal. (21 years old, Bogotá)

It should be noted that despite their anxieties regarding ill-treatment at health facilities, the women in this study all ended up at Oriéntame to confirm the completion of their abortion. We attribute this to Oriéntame seeming to be a safer way to enter the formal healthcare system for this group. In fact, at the time this study was conducted, the postabortion check-up service was unique to this provider, and advertisements for the service acknowledged that women may have used alternative methods to self-manage their abortions, which might have helped the participants in this study feel less stigmatized. Furthermore, participants noted that word of mouth from friends, relatives, and even the sellers from whom they obtained the medications helped assure the women that they would receive supportive care at this clinic.

### Reasons to buy misoprostol outside formal healthcare facilities

Participants related that they looked for misoprostol informally because of perceived benefits of using this medication and their preferences for their abortion experience.

#### Ease of access/privacy

Some women reported that they had wanted to resolve their pregnancies as quickly as possible, and that to do so, they needed an abortion option that was relatively easier to access and would not present as many delays for their process. When they learned of misoprostol either through their own experience or research, or from other sources, it appeared to them to be a method of pregnancy termination that could satisfy these needs.

…I started to get scared [when I realized I was pregnant] and I searched online. Something I had once heard was that a friend had aborted with Cytotec [a brand name of misoprostol], and that she got it online. …I searched the internet and it appear[ed], super easy. …you simply Google “Cytotec” and it appears on the first page …and they tell more or less about people’s experiences, testimonials… so I said, “Well, if it worked for my friend, it should work for me too.” (23 years old, Bogotá).

This desire to quickly identify a solution meant that some participants sought out misoprostol because they expected that terminating their pregnancies through the formal healthcare system would be a more protracted process.

Interviewer: Did you look into the possibility at some point of seeking care at a health institution?

Participant: Yes, but the process took longer…when you’re in trouble [and] the decision is made, you want to get out of it quickly. (34 years old, the Coffee Axis)

Social attitudes toward abortion influenced participants (and/or the individuals in whom they confided) to search for more private ways to both access and undergo an abortion. Though most participants obtained misoprostol themselves from drug shops, online, or street sellers, these were not very involved or lengthy transactions given the clandestine nature of the sale.

…I called a number that [friend] gave me…[friend] has had abortions like that in that way, so that’s why she told me that this was the best option….so that no one would find out…and well, I really don’t want…I mean, I don’t want anyone in the house, in my family, to find out. (23 years old, Bogotá)

#### Effectiveness and avoidance of invasive procedure

Another motivator cited by participants was the potential effectiveness of misoprostol. Three of the women in this study had previously used misoprostol, but others related feeling encouraged to use the medication after hearing of its efficacy from other people who had used it, as well as from those who sold them the medications informally. Some participants learned of these sellers through previous customers who had successfully aborted using the misoprostol purchased from them. Referrals like these built further confidence in both the seller and the efficacy of the pills.

Because I feared being pregnant - I do not have the conditions to have another child - a friend recommended to take some pills called Cytotec. She told me that they were effective, so I immediately looked for someone to lend me the money… and I went to a pharmacy and bought them… The man who sold [the pills] to me told me they were totally effective, so I trusted that, and I took them and waited for the result. (37 years old, Bogotá)

Along with effectiveness, a few participants noted that misoprostol allowed them to avoid a more involved procedure. These women thought that the legal process to have an abortion implied being hospitalized or needing to undergo a surgical method, which made using a medication like misoprostol their preferred option.

I was afraid of surgical intervention… I’m afraid of surgeries. I thought the pills were the best, most viable, and faster option. (37 years old, Bogotá).

#### Affordability

In comparison to legal abortion care, misoprostol purchased from informal sellers sometimes came to less than half the cost, making it a far more accessible option. In fact, participants often reported financial constraints as preventing them from obtaining legal abortion care. Some women lacked health insurance coverage because they were unemployed or had limited finances. While some participants assumed abortion care at a health center would be beyond their means, others contacted private providers in the hopes that it might fit into their budgets, only to find that the cost was out of reach.

I was told [by the facility] that I had to pay 480,000 Colombian pesos (COP) (approximately 150 United States dollars (USD) at the time we conducted this interview) [for abortion care]. The father of the baby did not have enough money, nor [did I know] anyone to ask for this sort of money. I had to pay for my child’s kindergarten, her food, - I spent all my salary on her. So, I told a friend that I definitely had to look for another alternative. She told me to call for the pills. (24 years old, Bogotá)

I looked for these pills because they were cheap. They told me, “They are worth COP 70,000 (approximately USD 21 at the time we conducted this interview).” I knew that at [a specific sexual and reproductive care clinic] it was a bit expensive, so I decided to do it on my own. And I said, “Well, it’s cheaper, and I’ll save money”. (26 years old, Bogotá)

For some women living outside of cities with private legal abortion providers such as Oriéntame, accessing a health center encompassed both the cost of care and the expense of traveling to another city. This put abortion care beyond their means or borrowing potential.

The thing is that the tickets [to travel to Bogotá by land] are more or less COP 100,000 (approximately USD 31 at the time we conducted this interview), the [abortion] procedure would cost me COP 507,000 (approximately USD 157 at the time we conducted this interview) [plus] medicines, stay, and food… so I had to get a million pesos (approximately USD 310 at the time we conducted this interview). As a student, recently graduated, who is not doing anything because unemployment is terrible in this country right now, how was I going to get a million pesos? It was simply impossible. (22 years old, Bogotá)

## Discussion

Our findings indicate that barriers to legal abortion care and the perceived benefits of self-managing motivated women to acquire abortion medications from informal sources. Barriers to obtaining legal abortion care included perceived and actual costs associated with abortion care; fear of judgment and stigmatization; and concerns about legal consequences. Conversely, the ease of access to misoprostol through informal sales, along with its affordability, and the greater opportunity for privacy afforded were greatly valued by the study participants. These findings align with a growing body of literature indicating that barriers to access discourage pregnant people from seeking legal abortion care, thereby increasing the likelihood that they will pursue informally self-managed abortion [30, 31].

Knowledge of the grounds for legal abortion care among women in our sample was, in most cases, inaccurate or nonexistent. This lack of clarity compounded other barriers to legal abortion care for this sample. When we conducted these interviews, abortion could have been accessible to many of the participants through the (mental) health exemption. However, knowledge of the permissible criteria for abortion was low; less than half of the participants were aware that there was a criterion based on health, and even fewer of this group considered the health exemption to also encompass mental health. Other work has found that the particularities of the legal framework of abortion are not only misinterpreted by those seeking abortion, but by health professionals as well. González Vélez and colleagues attribute this confusion to the continued criminal status of abortion following the 2006 decision [32]. While the health exemption was broad in its scope, abortion was still perceived as a crime and known as such [32]. Consequently, this positioned physicians to have a considerable role in determining whether an abortion request qualified as valid, a risk that participants in this sample feared.

Still, knowledge of the legal status of abortion would not necessarily have removed barriers to care for this sample. There is evidence that health care providers not only denied abortion requests but denied providing referrals as well [10, 11]. In fact, between 2006 and 2022, women seeking abortion would often have to go through the courts to submit an *acción de tutela*, a legal mechanism to obtain protection of fundamental rights, in order to secure their access to an abortion [33, 34]. Further, abortion providers have not been widely available across all territories, resulting in differential levels of accessibility to services [10]. 

Along with misinterpretation of the circumstances under which abortion could be accessed in 2018, women in our sample perceived legal abortion as a compromise to their privacy and put them at risk of being stigmatized. Participants believed that the inclusion of their abortions in their clinical records could lead to rejection from other healthcare providers and other personnel accessing these records.

They also perceived the healthcare system as a law enforcement actor, capable of imposing restrictions on their reproductive autonomy and forcing them into motherhood. In contrast, they found that buying the medication through an informal sale was a more accessible alternative that afforded them more privacy and reduced their risk of legal exposure. These findings echo those of Chemlal and Russo’s systematic review regarding the considerations of women who seek abortion through informal channels [35]. The authors argue that women consider health facilities unsafe spaces because they fail to protect the social reputation of their patients and leave them open to potential mistreatment from facility staff [35]. In fact, they found that women valued having their privacy maintained more highly than their physical health [35]. The ability of the healthcare system to maintain patient privacy may be particularly resonant in a country like Colombia, where health professionals are known to, and often, report women seeking postabortion care to the police [14]. Expecting to be subject to judgment and stigma when seeking abortion care ultimately undermines women’s trust in the healthcare system, while fear of exposure prevents them from engaging with the system at all.

Informal self-management with misoprostol was perceived by the women in this study as an easier, faster, and more private alternative for abortion. They also found misoprostol as a means of abortion to be far less expensive than private clinics, which was an especially important consideration for participants lacking health insurance coverage. Along with being a more affordable alternative for them, they also felt confident in the effectiveness of misoprostol as an abortifacient, either due to their own experience or through word of mouth. These findings echo other work exploring motivations to use medication abortion, particularly how informal self-management can minimize logistical considerations such as the need for travel, or more protracted processes within the formal medical system [30, 31]. In a systematic review on the various methods women have used for self-managed abortion, Moseson and colleagues note that women cite simplicity and privacy as reasons for using methods like medication abortion [36]. Self-managed abortion may reduce the burden of abortion stigma and allow pregnant people to avoid barriers to legal abortion care, while also empowering them to have more agency in their abortion experience [36]. Despite these positives, the experience of self-managed abortion can be adversely affected by having inadequate support or information, or when it is employed reactively because there do not seem to be any other alternatives to care. This can result in longer pathways to pregnancy termination, incomplete procedures, and otherwise negative abortion experiences [22, 36]. This spectrum of experiences highlights how crucial adequate information, effective medications, social support, and access to emergency care are to the experience of self-managed abortion.

### Importance of results

In 2022, the abortion law in Colombia was changed by the Constitutional Court through ruling C-055. Though the health, rape, and fetal malformation grounds for legal abortion under the previous abortion law were maintained for abortions after 24 weeks, all abortions occurring before this gestational age limit were now accessible on demand. This more liberal law may mitigate some of the challenges to accessing abortion care as it makes clear that no one having an abortion before 24 weeks of pregnancy should be subject to criminal charges. Our results highlight that an active law is not necessarily a well-known or well-understood law, however. At the time of this study’s interviews, the (previous) abortion law had been active for twelve years. Despite this lengthy timeframe, the women in this study displayed a limited awareness of the law and its parameters. There was a robust jurisprudence for access to abortion care, but the lack of knowledge of the legal framework among Colombia’s population prevented women from being able to exercise their rights [32]. This suggests that increasing the general public’s knowledge of the abortion law would be key to improving access to care. Still, while incomplete knowledge of both the law and availability of abortion services informed the decisions of women in this study to seek misoprostol, this was not the only factor. Abortion stigma, structural barriers, and fear of legal consequences also served as major obstacles to these women obtaining abortion in the formal healthcare system.

### Study limitations

This study has some limitations to consider. This was a small qualitative data collection effort that took place in two cities in Colombia. The perspectives of the group of women we interviewed allowed us to explore some of the motivations for obtaining misoprostol informally, but these experiences cannot be generalized to all women who obtain medication in this fashion. In fact, this work only captures insights among women who were willing to visit a private clinic to confirm the completion of their abortion attempts and may not represent women who do not seek or cannot afford this type of postabortion care. It also excludes the experiences of individuals whose self-managed abortions occurred with the support of an accompaniment group. Further, this study was primarily focused on exploring women’s experiences of obtaining and using informally acquired medications to self-manage. As a result, the interview guide was designed to delve more into this aspect of their experience rather than the decision-making that led to it.

### Further directions

These results indicate there was ambiguity in the interpretation and awareness of the 2006 abortion law. Now that the law has changed with the 2022 ruling, it would be worthwhile to explore awareness of the current law and how women in Colombia navigate access to legal services presently. There has been an observed increase in the number of women requesting use of health insurance to cover their first-trimester abortion care since the law changed [37]. Yet recent evidence also suggests that women still face obstacles to access, including health personnel imposing barriers on women seeking abortion care [37]. Given the role of health professionals in permitting or prohibiting access to abortion services, understanding their interpretation of the current law and their perceptions of their role in enacting it would also elucidate how and why health professionals may perpetuate barriers to care.

The fear of legal ramifications also represented a main consideration for many women in this study. Given the history of provider involvement in the prosecution of abortion [14], this highlights a need for future research to understand how health professionals and law enforcement currently approach reporting and prosecution (respectively) of abortion attempts both up to and after 24 weeks of pregnancy.

The more liberal abortion law may have changed the landscape in which informal sellers of abortion medications operate, although their services likely continue to be in demand. How informal sellers have modified their practices, if at all, in response to the law, is yet another means of understanding how the ramifications of the new law have impacted women’s access to abortion.

## Conclusions

Knowing more about women’s perceptions of the abortion care provided in formal healthcare settings deepens our understanding of their considerations when they undertake alternative pathways to abortion care. Fear of judgment, being reported to legal authorities, experiencing delays in care, and facing high costs for care discourage women from seeking abortion care at health facilities. It also provides insight into what may have made obtaining misoprostol through informal sales an appealing prospect prior to the full decriminalization of abortion up to 24 weeks. If women are to be supported in their abortion decisions, either in formal settings or after they have obtained the procedure in other ways, then strategies need to be implemented to improve awareness of the abortion law and its grounds among both potential users and providers of healthcare. There also needs to be proactive efforts to combat abortion stigma, which not only suppresses open dialogue on this topic, but impacts abortion decision-making and quality of care. This would not only support pregnant individuals knowing their rights and being able to fully exercise them, but also ensure that the principle of the law is executed in the day-to-day practice of legal abortion care.

### Supplementary Information

Supplementary Material 1.

## Data Availability

The dataset generated and analyzed during the current study is not publicly available due to potential confidentiality concerns and the sensitive nature of the topics addressed in these interviews. Additional information about these data can be made available upon reasonable request.

## Abbreviations

COP: Colombian pesos
USD: United States dollars
